# Effect of Mavacamten Therapy on Myocardial Systolic Function Assessed by 3D Echocardiography

**DOI:** 10.1016/j.jaccas.2026.107035

**Published:** 2026-03-04

**Authors:** Roberto Polizzi, Grazia Canciello, Leopoldo Ordine, Raffaella Lombardi, Giovanni Esposito, Maria Angela Losi

**Affiliations:** Department of Advanced Biomedical Sciences, University Federico II of Naples, Naples, Italy

**Keywords:** cardiomyopathy, echocardiography, treatment

## Abstract

**Background:**

Myosin inhibitors represent a novel therapeutic strategy for obstructive hypertrophic cardiomyopathy (oHCM), directly targeting sarcomeric hypercontractility to reduce left ventricular outflow tract obstruction.

**Case Summary:**

Three patients with symptomatic oHCM enrolled in Italy's Early Access Program for mavacamten underwent 2D and 3D echocardiographic assessment at baseline and at 6 months. All experienced symptomatic improvement and left ventricular outflow tract obstruction reduction, accompanied by a modest decline in global left ventricular ejection fraction (LVEF) at 6 months follow-up. A 3-dimensional analysis revealed that LVEF reduction primarily involved mid and apical segments, while the basal function remained stable.

**Discussion:**

These findings suggest that mavacamten selectively attenuates hypercontractility in relatively preserved myocardial regions rather than inducing global systolic dysfunction. Three-dimensional echocardiography provided novel insights into regional contractile adaptation, offering potential guidance for individualized dosing and monitoring.

**Take-Home Messages:**

Mavacamten-induced LVEF decline reflects regional hypokinesia, not global dysfunction. Three-dimensional imaging enhances understanding of treatment response in oHCM.

## Visual Summary

**Figure:**
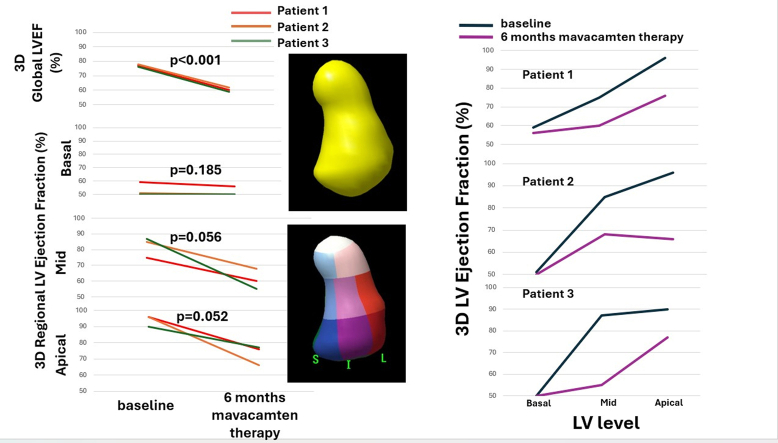

**Visual Summary:**
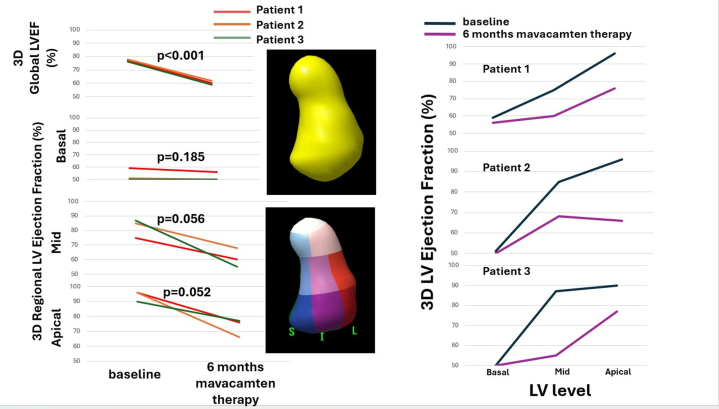
Effect of Mavacamten on Global and Regional Left Ventricular Ejection Fraction Changes of left ventricular global and regional left ventricular ejection fraction assesses by 3D at baseline and after 6 months of mavacamten therapy. LV = left ventricular; LVEF = left ventricular ejection fraction.

Myosin inhibitors have recently emerged as a therapeutic option for symptomatic obstructive hypertrophic cardiomyopathy (oHCM), providing a targeted approach to reducing left ventricular (LV) outflow tract obstruction (LVOTO) by directly modulating sarcomeric contractility.^1^ In contrast to traditional pharmacological agents, such as beta-blockers, nondihydropyridine calcium channel blockers, and disopyramide, which reduce obstruction through indirect effects on heart rate or loading conditions, myosin inhibitors decrease the formation of actin–myosin cross-bridges, thereby specifically counteracting the underlying hypercontractile state characteristic of hypertrophic cardiomyopathy (HCM).^2^

**Take-Home Messages**

• Mavacamten-induced left ventricular ejection fraction reduction reflects regional normalization of hypercontractility, not diffuse systolic dysfunction.
• Three-dimensional echocardiography provides critical insights for individualized dosing and safe optimization of myosin inhibitor therapy in obstructive hypertrophic cardiomyopathy.

Clinical trials and data from the real word have shown that mavacamten, the first agent of this class approved for clinical use, can improve symptoms, exercise tolerance, and quality of life in patients who remain symptomatic despite guideline-directed medical therapy.^3^, ^4^, ^5^ These benefits are accompanied by significant reductions in LVOTO at rest and with provocation, supporting the role of myosin inhibition in modifying disease physiology rather than simply alleviating symptoms.

However, the potential reduction in LV ejection fraction (LVEF) observed during treatment remains a key consideration^6^ although this effect is generally reversible with dose adjustment or temporary drug suspension. Importantly, in HCM, myocardial hypertrophy and fibrosis are often regionally distributed, and hypercontractility may be exaggerated in specific nonhypertrophied segments that compensate for structurally abnormal regions. Therefore, a decline in LVEF may not necessarily reflect a global impairment of systolic function but could instead represent a rebalancing of regional contractile patterns.

Three-dimensional echocardiography offers the potential to clarify these effects by enabling more accurate assessment of LV segmental contributions to global systolic performance.^7^ By providing a more comprehensive view of myocardial mechanics, 3D imaging may help distinguish between therapeutic normalization of contractility and systolic dysfunction. This information could be crucial in guiding dose titration and ensuring both the efficacy and safety of myosin inhibitor therapy in patients with obstructive HCM.

## Case Series

Italy launched an Early Access Program (EAP) (Camzyos). During the limited timeframe in which our center was able to participate, we enrolled 3 patients with symptomatic oHCM who remained limited by exertional or postprandial symptoms despite maximal tolerated medical therapy. All patients underwent comprehensive evaluation including 2D and 3D echocardiography. The 3D full-volume technique allowed assessment of regional LVEF and comparison of segmental systolic performance from baseline to 6 months.^7^ All patients provided informed consent for participation. Paired *t*-test was performed to check differences between baseline and 6-month follow-up in 3D global and regional LVEF.

### Patient 1

A 76-year-old woman with no family history of sudden death or cardiomyopathy presented 3 months after newly detected severe LV hypertrophy. She was obese (Body Mass Index = 31), had dyslipidemia on statins, and no diabetes, hypertension, or syncope. She also reported recent dysuria. At presentation, she was NYHA class III (Table 1).

**Table 1:** Baseline Characteristics of the Three Cases

Case	Age (y)	Sex	NYHA Functional Class	Postprandial Symptoms	Therapy Die	MWT (mm)	Location MWT	LVOTO Rest (mm Hg)	LVOTO Valsalva (mm Hg)	LVEF (%)
1	76	f	III	No	Metoprolol 50 mg × 2	27	Basal septum	86	90	63
2	54	m	II	Yes	Metoprolol 50 mg × 2	20	Basal septum	18	86	66
3	32	m	II	Yes	Metoprolol 100 mg × 2; disopyramide 250 mg × 2	27	Basal septum	50	70	72

f = female; LVEF = left ventricular ejection fraction; LVOTO = left ventricular outflow tract obstruction; m = male; MWT = maximal wall thickness; NYHA = New York Heart Association.

**Figure 1:**
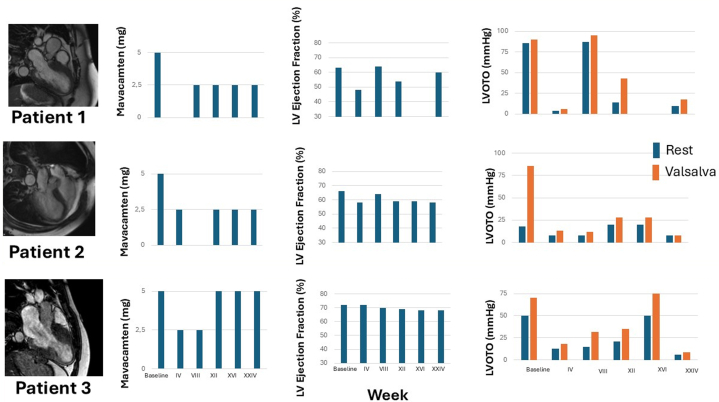
Mavacamten Dosage, Left Ventricular Ejection Fraction and Left Ventricular Outflow Tract Obbstruction From Baseline to the XXIV Week of Treatment Changes throughout follow-up of mavacamten dosage, 2D left ventricular ejection fraction, and left ventricular outflow tract obstruction either at rest and during Valsalva maneuver in the 3 cases. LV = left ventricular; LVOTO = left ventricular outflow tract obstruction

Cardiac magnetic resonance imaging (MRI) showed a maximal basal septal thickness of 24 mm (Figure 1, upper left), diffuse nonischemic late gadolinium enhancement (LGE), and LVEF 82%. Light chain amyloidosis and wild-type transthyretin amyloidosis and HCM-related genetic mutations were excluded, leading to a diagnosis of nonsarcomeric HCM. Echocardiography revealed severe LVOTO from systolic anterior motion (SAM), with a resting gradient of 86 mm Hg and 90 mm Hg after Valsalva, and LVEF 63% (Table 1).

Metoprolol was started and increased to 100 mg/day, but symptoms persisted (Table 1); dysuria contraindicated disopyramide, so she entered the EAP for mavacamten, with metoprolol reduced to 50 mg/day. The upper panel of Figure 1 shows changes in mavacamten dose, LVEF, and LVOTO over 6 months. At 6-month follow-up, she was stable on mavacamten 2.5 mg/day, LVEF was 60%, and LVOTO had fallen to <30 mm Hg at rest and with Valsalva. Clinically, she improved to NYHA class II with intermittent complete symptom relief.

Three-dimensional echocardiography was performed at baseline and 6 months. Global 3D LVEF decreased from 78% to 60%. Segmental LVEF at baseline and follow-up was 59%→56% (basal), 75%→61% (mid), and 96%→77% (apical) (Central Illustration, bottom left, and upper right panels).

### Patient 2

A 53-year-old man with family history of sudden death and HCM, with MYBPC3 sarcomeric mutation, implanted with an intracardiac defibrillator that successfully resuscitated a sudden cardiac arrest 1 year earlier, and regularly followed up to our outpatient clinic for HCM referred worsening of symptoms in the last 6 months, reaching a NYHA class III. He was not obese (BMI 26) and had no dyslipidemia, diabetes, or hypertension (Table 1).

Cardiac MRI performed 2 years before the present report showed a maximal basal septal thickness of 22 mm (Figure 1, mid left), nonischemic LGE at right ventricular insertion to ventricular septum and at the antero-lateral wall level, and an LVEF of 68%. In the last year, because of LVOTO and symptoms, metoprolol (200 mg/day) had been initiated and later added to disopyramide, which, however, was withdrawn because of erectile dysfunction development regressed after discontinuation of the drug (Table 1). Echocardiography revealed severe LVOTO from SAM, with a resting gradient of 16 mm Hg and 86 mm Hg after Valsalva and LVEF of 66% (Table 1), so he entered the EAP for mavacamten, with metoprolol reduced to 50 mg/day. The mid panels of Figure 1 shows changes in mavacamten dose, LVEF, and LVOTO over 6 months. At 6-month follow-up, he was stable on mavacamten 2.5 mg/day, LVEF was 58%, and LVOTO had fallen to <30 mm Hg at rest and at Valsalva maneuver. Clinically, he improved to NYHA class II with intermittent complete symptom relief.

Three-dimensional echocardiography was performed at baseline and 6 months. Global 3D LVEF decreased from 78% to 62%. Segmental LVEF at baseline and follow-up was 51%→50% (basal), 85%→68% (mid), and 97%→67% (apical) (Central illustration, mid-left, and mid-right panels).

### Patient 3

A 32-year-old man with a family history of sarcomeric HCM, with MYH7 sarcomeric mutation, implanted with an implantable cardioverter-defibrillator for primary prevention had been regularly followed up to our outpatient clinic in the last 2 years. He was obese (BMI 31) and had no dyslipidemia, diabetes, or hypertension. Cardiac MRI performed 1 year before the present report showed a maximal basal septal thickness of 23 mm (Figure 1, bottom left), nonischemic LGE at the antero-lateral wall level, and an LVEF of 76% (Table 1). Because of symptoms and significant LVOTO, he has been treated with metoprolol 200 mg/die and disopyramide 500 mg/die. However, he referred further worsening of postprandial symptoms, like palpitations, angina, and dyspnea, with no amelioration of functional class (NYHA class II) (Table 1). Echocardiography revealed severe LVOTO from SAM, with a resting gradient of 50 mm Hg and 70 mm Hg after Valsalva, and LVEF of 72% (Table 1), so he entered the EAP for mavacamten. Disopyramide was discontinued. The bottom panel of Figure 1 shows changes in mavacamten dose, LVEF, and LVOTO over 6 months. At 6-month follow-up, he was stable on mavacamten 5 mg/day, LVEF was 68%, and LVOTO had fallen to <30 mm Hg at rest and at Valsalva maneuver. Clinically, he was stable in NYHA class II with complete relief of postprandial symptoms.

Three-dimensional echocardiography was performed at baseline and 6 months. Global 3D LVEF decreased from 78% to 62%. Segmental LVEF at baseline and follow-up was 50%→50% (basal), 87%→55% (mid), and 90%→77% (apical) (Video 1, Video 2).

## Discussion

Our observations suggest that the reduction in LVEF associated with mavacamten therapy is not uniformly distributed throughout the LV, but instead predominantly affects regions that are relatively hyperdynamic at baseline. This is particularly relevant in HCM, in which both the myopathic process and the degree of hypertrophy are heterogeneously distributed. Hypertrophied septal segments are known to demonstrate increased stiffness, impaired relaxation, microvascular ischemia, and intrinsically reduced systolic wall motion, whereas adjacent nonhypertrophied regions often display compensatory hypercontractility.^8^

It is also important to acknowledge that the apparent gradient in systolic performance from base to apex is influenced not only by a true physiological pattern, where basal segments normally exhibit less systolic excursion than mid and apical regions, but also by intrinsic limitations of conventional cardiac imaging.^9^ Both echocardiography and cardiac magnetic resonance may underestimate basal contraction due to tethering effects, through-plane motion, and geometrical interactions between the basal myocardium and the mitral annulus.^9^ As a result, basal regional LVEF frequently appears lower than its actual contribution to forward stroke volume. Nevertheless, even accounting for these technical factors, some degree of basal systolic change would still be expected when pharmacologically reducing actin–myosin cross-bridging.

Thus, the fact that basal segmental systolic performance remained essentially unchanged during therapy in our patients, while mid and apical contractility declined, is therefore notable and cannot be attributed solely to imaging artifact. In this context, our findings support the interpretation that mavacamten primarily attenuates excessive systolic performance in relatively preserved, nonhypertrophied myocardial regions, while exerting minimal further impairment in hypertrophied segments that are already functionally limited by structural disarray, fibrosis, and microvascular dysfunction. Such selective reduction in contractility is consistent with the drug's mechanism of action, namely decreasing the number of actin–myosin cross-bridges available during systole and thereby normalizing the contractile reserve in myocardial regions exhibiting augmented function at baseline.^3^

These observations raise the possibility that the decrease in global LVEF observed with mavacamten may represent a redistribution or normalization of regional systolic performance rather than a diffuse systolic impairment. From a pathophysiological standpoint, attenuating hypercontractility in nonhypertrophied or less-hypertrophied regions than septum, LV segments may reduce regional myocardial stress and oxygen demand, potentially moderating the drivers of adverse remodeling over time. Given that hyperdynamic segmental contraction in HCM often serves as a compensatory mechanism, its pharmacological modulation may shift the ventricle toward a more energetically efficient pattern of systolic function.^10^

Three-dimensional echocardiography therefore provides valuable insight into these regional mechanical effects and offers a more refined means of evaluating treatment response in HCM than global LVEF alone. Systematic regional assessment may also help guide dose titration and identify patients who exhibit excessive reductions in contractile function. More broadly, integrating regional functional analysis into therapeutic monitoring could enhance patient selection and support a more individualized approach to myosin inhibitor therapy.

## Conclusions

In this small case series, mavacamten therapy in patients with symptomatic oHCM resulted in symptomatic improvement and marked LVOTO reduction, accompanied by a modest decline in global LVEF at 6 months of follow-up. The 3-dimensional echocardiographic analysis revealed that this reduction primarily affected mid and apical regions, while basal function remained unchanged, suggesting a selective attenuation of hypercontractility in nonhypertrophied myocardial segments. These findings support the concept that myosin inhibition leads to a regional rebalancing of systolic function rather than global systolic depression, highlighting the importance of advanced imaging in monitoring therapeutic effects.

## Funding Support and Author Disclosures

The authors have reported that they have no relationships relevant to the contents of this paper to disclose.

## Appendix

Video 1 Three-Dimensional Full Volume of Patient 3 at Baseline

Video 2 Three-Dimensional Full Volume of Patient 3 After 6 Months of Follow-Up
